# The heat is on: a simple method to increase genome editing efficiency in plants

**DOI:** 10.1186/s12870-022-03519-7

**Published:** 2022-03-24

**Authors:** Jonas Blomme, Ward Develtere, Ayse Köse, Júlia Arraiza Ribera, Christophe Brugmans, Jessica Jaraba-Wallace, Ward Decaestecker, Debbie Rombaut, Alexandra Baekelandt, Álvaro Daniel Fernández Fernández, Frank Van Breusegem, Dirk Inzé, Thomas Jacobs

**Affiliations:** 1 Department of Plant Biotechnology and Bioinformatics, Ghent University, 9052 Ghent, Belgium; 2 VIB Center for Plant Systems Biology, 9052 Ghent, Belgium; 3 Phycology Research Group, Department of Biology, Ghent University, 9000 Ghent, Belgium; 4 Bioengineering Department, Ege University, 35100 Izmir, Turkey

**Keywords:** CRISPR, Plant biotechnology, Genome engineering, Heat-shock, Base editing, Homology-directed repair

## Abstract

**Background:**

Precision genome mutagenesis using CRISPR/Cas has become the standard method to generate mutant plant lines. Several improvements have been made to increase mutagenesis efficiency, either through vector optimisation or the application of heat stress.

**Results:**

Here, we present a simplified heat stress assay that can be completed in six days using commonly-available laboratory equipment. We show that three heat shocks (3xHS) efficiently increases indel efficiency of LbCas12a and Cas9, irrespective of the target sequence or the promoter used to express the nuclease. The generated indels are primarily somatic, but for three out of five targets we demonstrate that up to 25% more biallelic mutations are transmitted to the progeny when heat is applied compared to non-heat controls. We also applied our heat treatment to lines containing CRISPR base editors and observed a 22-27% increase in the percentage of C-to-T base editing. Furthermore, we test the effect of 3xHS on generating large deletions and a homologous recombination reporter. Interestingly, we observed no positive effect of 3xHS treatment on either approach using our conditions.

**Conclusions:**

Together, our experiments show that heat treatment is consistently effective at increasing the number of somatic mutations using many CRISPR approaches in plants and in some cases can increase the recovery of mutant progeny.

**Supplementary Information:**

The online version contains supplementary material available at 10.1186/s12870-022-03519-7.

## Background

Programmable site-specific genome mutagenesis using CRISPR/Cas is a powerful technique that has been widely adopted, adapted and improved by biologists since the first report [1]. CRISPR has been successfully applied in a number of plant species including important crops by delivering a nuclease such as Cas9 or Cas12a paired with a guide RNA (gRNA) complementary to a DNA target site in the host genome (reviewed in [2–4]). In its most common use, the gRNA determines where a double-stranded DNA break (DSB) is made. The DSB is repaired by the cell’s machinery, but this process is error-prone and can generate insertions or deletions (indels) at the target site. When an exogenous donor template sequence is provided, homology-directed repair (HDR) pathways can integrate the template at the DSB site [5]. Alternatively, deactivated nucleases can be fused to various DNA-modifying domains to create precise base edits [6, 7].

One of the challenges of generating knock-out plant lines is the generation of alleles that are transmitted to the progeny through the germline. Several adaptations to transformation vectors have been used to increase genome editing efficiency, such as using different constitutive or germline-specific promoters to control *Cas9* expression [8–13] or optimization of other regulatory sequences such as terminators, introns or U6 promoters that drive gRNA expression [14].

Next to optimizing transformation vectors, mutagenesis efficiency can be increased by applying small molecules in mammalian cells [15, 16] or applying heat [17–21]. Human cell lines incubated at high temperatures (33, 37 and 39 °C) three days after transfection with Cas9 ribonucleoproteins (RNPs) display more indels compared to cell lines incubated in hypothermic conditions (30 °C; [21]). In zebrafish, higher indel rates with *Acidaminococcus* (As)Cas12a and *Lachnospiraceae bacterium* (Lb)Cas12a RNPs, but not Cas9 RNPs, were observed when embryos were incubated at 34 °C versus 28 °C for 4-24 h after transfection [19]. Similarly, heat-shocking cells of the green algae *Chlamydomonas reinhardtii* before delivery of Cas9 RNPs increases indel efficiencies 15-fold [22]. In Arabidopsis, four 30 h heat cycles (37 °C) alternated with 42 h recovery (22 °C) on two-week-old transgenic lines expressing Cas9 under the control of the YAO promoter increases in somatic indels ~5-fold [17]. A similar increase in mutations was observed in *Citrus* containing YAO-driven Cas9 exposed to seven 37 °C heat cycles [17]. Furthermore, cultivating rice cells or Arabidopsis Cas12a lines at 28 or 29 °C versus 22 °C can boost activity without additional heat shock treatment [18]. Similarly, applying heat treatment enhances mutagenesis in cotton (LbCas12a; [23]), poplar (AsCas12a; [24]) and wheat (Cas9; [25]). In Arabidopsis, a single 24 h 37 °C heat treatment is sufficient to increase mutation frequency of SpCas9 [26]. Other reports in some animal, algae and plant systems combine a heat-shock inducible promoter controlling *Cas9* expression with an incubation time at higher temperature after transfection [22, 27–29]. Though, as these are mainly used as inducible systems for transgene expression, it is difficult to separate the inducible expression of the nuclease from the specific impact of a heat-shock treatment. Altogether, these reports indicate that exposing cells or organisms to a heat shock or warmer temperatures can increase the efficiency of CRISPR mutagenesis systems.

The underlying cause of increased mutagenesis with CRISPR nucleases at higher temperatures has mainly been explained by an increase in nuclease and gRNA activity. *In vitro* activity of Cas9 and Cas12a and *in vivo* LbCas12a and gRNA expression is positively affected by higher temperatures, but Cas9 expression is unaffected [17, 21, 23, 26, 30]. An *in vitro* cleavage study illustrated an optimal activity of AsCas12a at 37 °C [30], although the optimal temperature in plant cells was reported to be ~28 °C [18]. Besides a higher on-target mutation rate, an increase in off-target mutation rates has also been observed [17, 21].

In this report, we further support the idea of using a heat treatment to increase the success of a variety of CRISPR experiments in plants. We present a simplified heat stress assay that can be completed in approximately one week using commonly-available laboratory equipment. Our results also demonstrate that base editing efficiency, but not HDR, can be increased with heat treatment. We observe a consistent increase in somatic indel frequencies using LbCas12a and Cas9 in *Arabidopsis thaliana* and *Nicotiana tabacum* and these increases are independent of the target gene and promoter sequence regulating Cas9 expression. Importantly, we were able to obtain an increase in the rate of inheritable mutations for three out of five gRNAs tested.

## Results

### Development of a heat stress assay

**Fig. 1:**
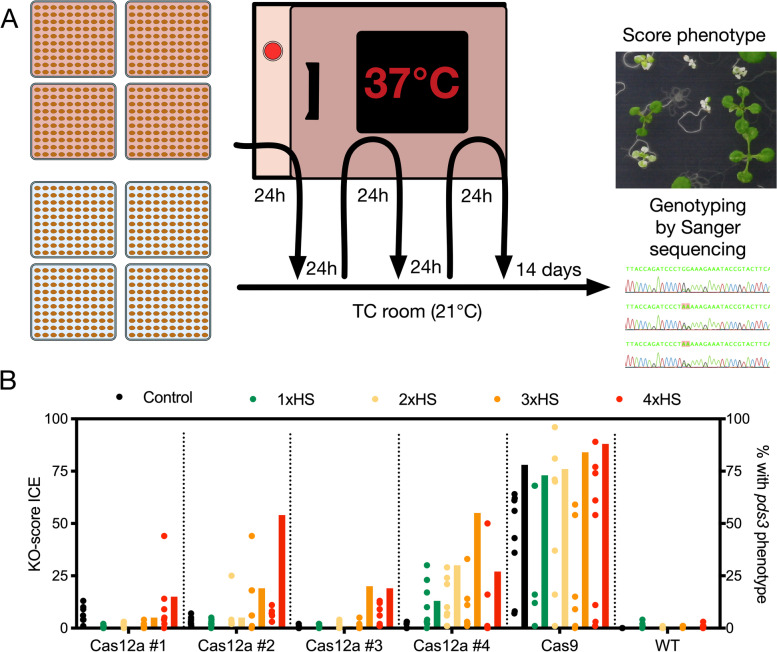
High temperature affects genome editing efficiency. **A** Heat shock protocol. Every line is either subjected to a series of three heat shocks (indicated with red squares) or is grown under normal conditions (21°C) in a Tissue Culture (TC) room. Each 24h heat shock at 37°C in a bacterial incubator is alternated with a 24h recovery period in a TC room (21°C). After the final heat shock, plants are allowed to recover and grow for ~14 days, followed by visual scoring of phenotypes (if possible) and/or genotyping by Sanger sequencing. **B** Effect of number of consecutive heat shocks on genome editing efficiency. Segregating Arabidopsis T2 lines expressing *PcUBI::LbCas12a::G7T* (four independent lines, Cas12a #1-#4) or *PcUBI::Cas9::G7T* and a gRNA targeting *PDS3* were grown under control conditions (21°C) or subjected to one, two, three or four heat shocks (1-4xHS; 37°C). After 14 days of recovery, plants displaying a *pds* phenotype were scored. *n*=75 per line per treatment (bars). DNA was extracted for eight randomly selected individuals for each line and treatment and PCR products amplified from targeted loci were sequenced and analysed using ICE (https://ice.synthego.com). The KO-score is given for each sample (dots), which indicates those indels that result in a frameshift or are 21+bp in length. *n*=8 per sample per treatment

We created transgenic Arabidopsis lines expressing three different Cas12a proteins (AsCas12a, *Francisella novicida* (Fn)Cas12a or LbCas12a) targeting *PHYOTENE DESATURASE 3* (*PDS3, AT4G14210*) with three individual CRISPR RNAs (crRNAs). Cas12a expression was controlled by the *Petroselinum crispum* Ubiquitin4-2 (PcUBI) promoter [31] and the G7 terminator and the crRNAs were controlled by the RPS5A promoter and RBCS terminator [32]. Individual crRNAs were flanked by the HH and HDV ribozymes [33]. Somatic mutations in *PDS3* cause white spots or mosaic sectors and biallelic knock-out results in dwarf albino plants ([13]; Fig. 1 A). None of the 190 T1 plants transformed with these Cas12a vectors displayed the expected *pds3* phenotype when grown under standard conditions. Inspired by previous reports, we imposed four cycles of 30 h at 37 °C (heat stress) and 42 h at 21 °C (recovery) on eight-day-old soil-grown segregating T2 plants using a Lovibond [17]. Using this set-up, we observed *pds3* phenotypes in lines containing LbCas12a and one of three crRNAs (Fig. S1A). Consistent with the phenotyping results, indel frequencies were at background levels in plants grown under control conditions whereas indel frequencies increased to up to 35% in individual plants when four heat shocks were applied to LbCas12a lines (Fig. S1B).

This experimental set-up makes use of dedicated growth chambers to apply heat stress [17, 18], which might not be regularly available to a wide range of laboratories. During our experiment, we also encountered uncontrolled fluctuations in temperature in the greenhouse and soil-grown plants were difficult to screen for *pds3* phenotypes (Fig. S1A). Taking these issues into account, we aimed to develop a simple and straightforward method to apply heat stress to increase indel rates. We challenged different Arabidopsis lines grown *in vitro* with one, two, three or four 24 h heat shocks (37 °C) in a bacterial incubator immediately after stratification. Each heat shock was alternated with 24 h recovery in a tissue culture room under standard growth conditions (21 °C; Fig. 1 A) and the entire heat treatment takes six days to complete. After the final heat shock, the seedlings were grown for 14 days, their phenotype scored and samples harvested for genotyping by Sanger sequencing (Fig. 1 A).

To develop this method, we selected segregating T2 transgenic Arabidopsis lines targeting *PDS3* with either the functional LbCas12a (described above) or Cas9 (*PcUBI::Cas9::G7T*) nucleases. Under control conditions (21 °C), Arabidopsis plants containing LbCas12a only very rarely (two out of ~13,000 plants) displayed a *pds3* phenotype whereas 78% of individuals containing Cas9 clearly displayed white sectors up to full albinos (Fig. 1B; Fig. S1B). In contrast, we observed clear white sectors in LbCas12a plants following three or four heat shocks (Fig. 1B; Fig. S1C). Depending on the line tested, these white sectors appeared in 5-55% of seedlings after three heat shocks (3xHS; Fig. 1B). This trend is less obvious in our positive control Cas9 plants, as the rate of *pds3* phenotypes are already high under control conditions (78% vs. 84% after 3xHS) and most likely saturating as the T2 lines are segregating for the T-DNA (Fig. 1B; Fig. S1C).

To confirm that an increase in *pds3* phenotypes was due to mutations in *PDS3*, DNA was extracted from eight randomly-selected T2 individuals per line and treatment and *PDS3* PCR products were Sanger sequenced and analysed with ICE (https://ice.synthego.com/#/). Consistent with the phenotyping results, indel frequencies were at background levels in individual plants grown under control conditions whereas average indel frequencies increased to 10-25% when heat shocks were applied to LbCas12a lines (Fig. 1B). These results confirm that even a single heat treatment can increase indel rates in certain Arabidopsis lines [26], but three or four heat shocks are most effective. Since some lines subjected to 4xHS recovered poorly from their final stress treatment (Fig. S2A), 3xHS was used for subsequent experiments.

To determine the optimal heat shock temperature, we imposed 3 × 30 °C, 3 × 37 °C or 3 × 42 °C treatments on two homozygous T3 LbCas12a transgenic lines. *pds3* phenotypes were only observed when plants were subjected to the 3 × 37 °C HS treatment and the 3 × 42 °C HS treatment was too severe for plants to germinate and survive (Fig. S2B). These results indicate that 37 °C is the optimal temperature for heat shock.

In addition to these heat shocks, we evaluated several other stress conditions to interrogate whether they could also induce indels. Segregating LbCas12a T2 plants were subjected to a variety of mild and severe genotoxic or abiotic stress conditions. The medium was supplemented with Mannitol (25mM or 50mM), NaCl (50mM or 100mM), Bleomycin (0,3 µg/mL) or Hydroxyurea (0,75mM). We treated the transgenic lines to a continuous low-dose UV stress (40 W/m^-2^ light supplemented with 0,42 W/m^-2^ UV) for one, two or three days. Although occasional single plants presented mild *pds3* phenotypes when subjected to osmotic or genotoxic stresses, the increase in the presence or severity of *pds3* phenotypes was not as pronounced as when the plants underwent a 3xHS (Fig. S3).

In conclusion, this set-up allows one to increase the production of indels with LbCas12a and Cas9 by applying a heat stress to Arabidopsis immediately after stratification with an effective temperature of 37 °C.

### Heat stress affects gene knockout irrespective of the transcriptional regulator

The transgenic lines we used to establish the heat-shock assay express Cas9 or LbCas12a with the PcUBI promoter. To investigate whether heat-induced indels are promoter-specific, we tested three commonly-used constitutive promoters, 35S, RPS5A and ZmUBI to drive Cas9 expression [34–36]. We screened 9-14 independent segregating T2 lines for each promoter-Cas9 combination, targeting *PDS3* with two to four different gRNAs. We observed an increase in the number and/or severity of *pds3* phenotypes after 3xHS treatment for ZmUBI-Cas9 (6/9 independent lines), RPS5A-Cas9 (14/14 independent lines), 35S-Cas9 (6/12 independent lines) and PcUBI-Cas9 (7/9 independent lines; Fig. S4). Although the phenotypes were relatively mild for 35S-Cas9 lines, our data are consistent with other reports that heat stress increases indel efficiencies independent of the promoter sequence used to drive Cas9 [17, 18, 26].

### Heat shock increases genome editing efficiency in Tobacco

**Fig. 2:**
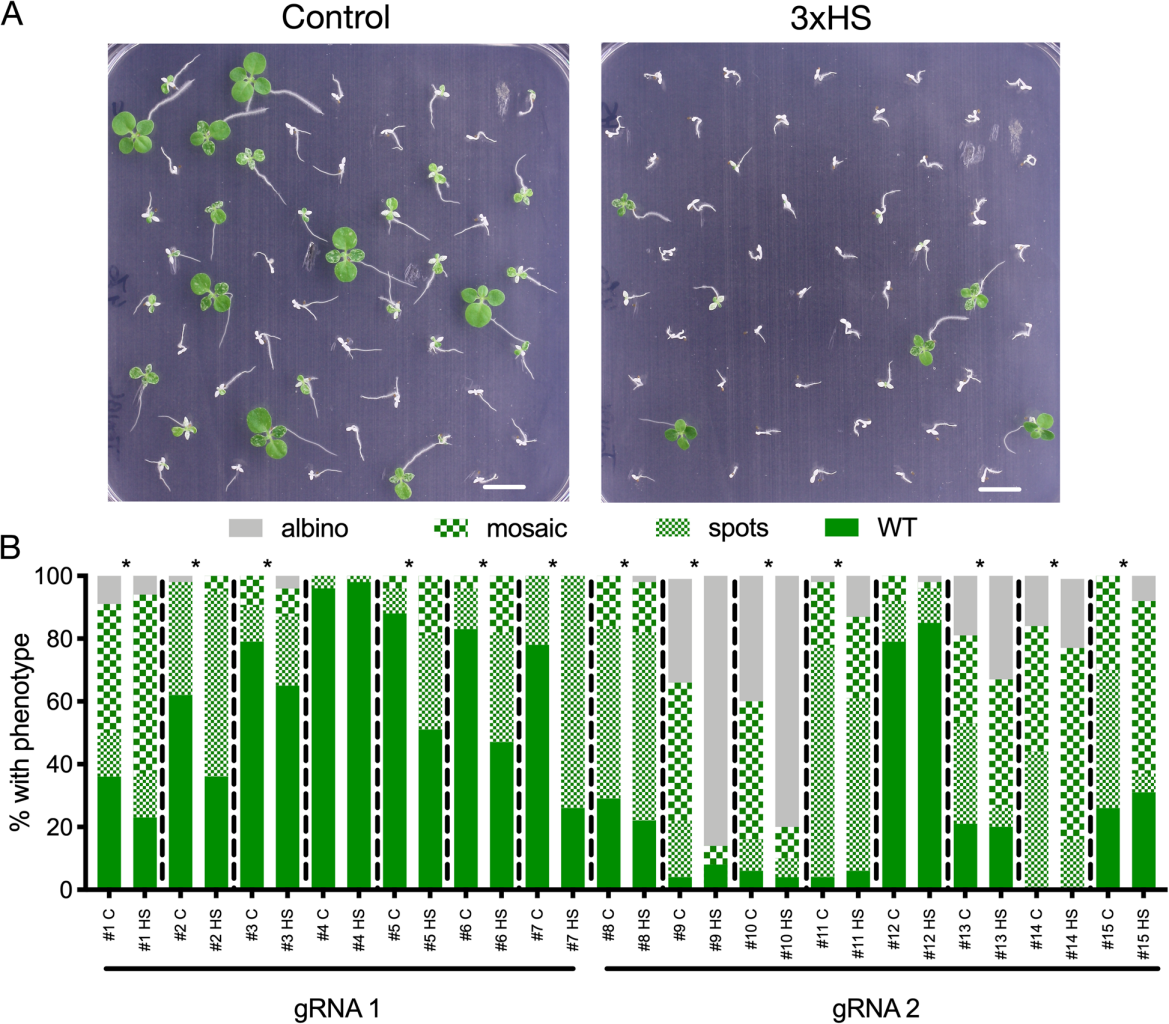
Heat stress increases genome editing efficiency in *Nicotiana tabacum*. **A** Phenotype of *in vitro *grown segregating *PcUBI::SpCas9::G7T* lines under control conditions or 3xHS regime. Picture taken 14 days after the last heat shock. Scale=1cm. **B** T2 segregating lines of *PcUBI::Cas9::Pea3AT *targeting *PDS3* with one of two gRNAs were grown under control conditions ﻿(C)﻿ or subjected to 3xHS (HS). *pds3*phenotypes were scored according to the severity (albino, mosaic or spots) 14 days after final HS for each line and condition. Lines that show an increase in number and/or severity of *pds3* phenotypes after 3xHS are indicated with *. *n*=50 for each line and condition

To demonstrate that our heat stress assay can increase indel production in other species, we investigated the effect of using 3xHS in Tobacco (*Nicotiana tabacum*) Cas9 segregating T1 lines again using the *PDS3* gene as a visual marker (Fig. 2 A). We observed an increase in the number and/or severity of *pds3* phenotypes after 3xHS treatment for six out of seven and seven out of eight independent lines for the two gRNAs used, respectively (Fig. 2B). These data confirm that heat stress can affect indel rates in multiple plant species, as also illustrated for *Citrus*, cotton, maize, poplar, rice and wheat [17, 18, 23–25].

### Heat stress induces genome editing irrespective of the target

**Fig. 3:**
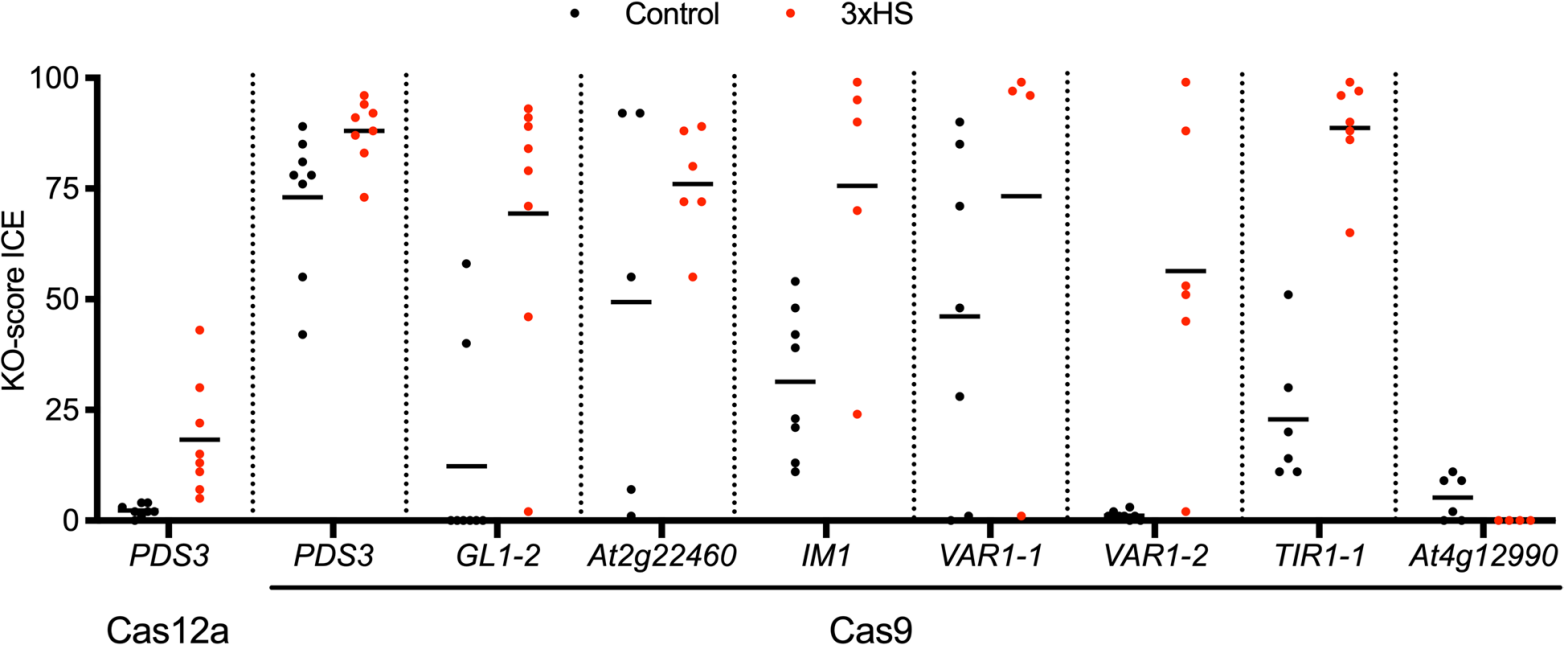
Indel efficiency increases after heat shock, irrespective of the target gene. T1 plants were used containing LbCas12a or Cas9 and targeting *PDS3*, *GLABRA1* (*GL1-2*), *At2g22460*, *IMMUTANS1* (*IM1*), *VARIEGATED1* (*VAR1-1* and *VAR1-2*), *TRANSPORT INHIBITOR RESPONSE 1 *(*TIR1-1*) or *At4g12990*. DNA was extracted for four to eight randomly selected individuals for each line and treatment, PCR products amplified from targeted loci were sequenced and analyzed using ICE (https://ice.synthego.com). The KO-score is given for each sample, indicating indels that result in a frameshift or are 21+bp in length. Lines indicate mean KO-score per sample per treatment. *n*=4-8 per sample per treatment

To determine if the increase in indel frequency observed for *PDS3* could be generalized to other gene targets, we selected T1 Arabidopsis lines expressing the same Cas9 or LbCas12a vectors targeting *PDS3* as well as seven additional Cas9 targets: *GLABRA1* (*GL1-2; AT3G27920*), *AT2G22460*, *IMMUTANS1* (*IM1; AT4G22260*), *VARIEGATED1-1* (*VAR1; AT5G42270*) and *VAR1-2*, *TRANSPORT INHIBITOR RESPONSE 1-1* (*TIR1-1; AT3G62980*) or *AT4G12990* (Fig. 3). All transformants were selected using a modified FAST system (*pOLE1::OLE1::mRuby3*; [37]) and subjected to either normal conditions or the 3XHS regime. Four to eight individuals per condition per target were randomly selected for DNA extraction and PCR products amplified from targeted loci were Sanger sequenced. We observed increased indel rates after a 3xHS regime for all targets except *AT4G12990* (Fig. 3). For *PDS3*, an average increase in indels of 16% and 15% was observed for lines containing LbCas12a and Cas9, respectively, confirming the results obtained in T2 lines (Figs. 1 and 3). For two targets, *VAR1-1* and *AT2G22460*, the average increase in indels was 27% upon 3xHS. For the four remaining targets, the effect of 3xHS was more pronounced: 44% for *IM-1*, 55% for *VAR1-2*, 57% for *GL1-2* and 66% for *TIR1*. These results suggest that our 3xHS treatment is robust to induce indels in Arabidopsis irrespective of the target sequence.

### Heat stress induces inheritable mutations

**Fig. 4:**
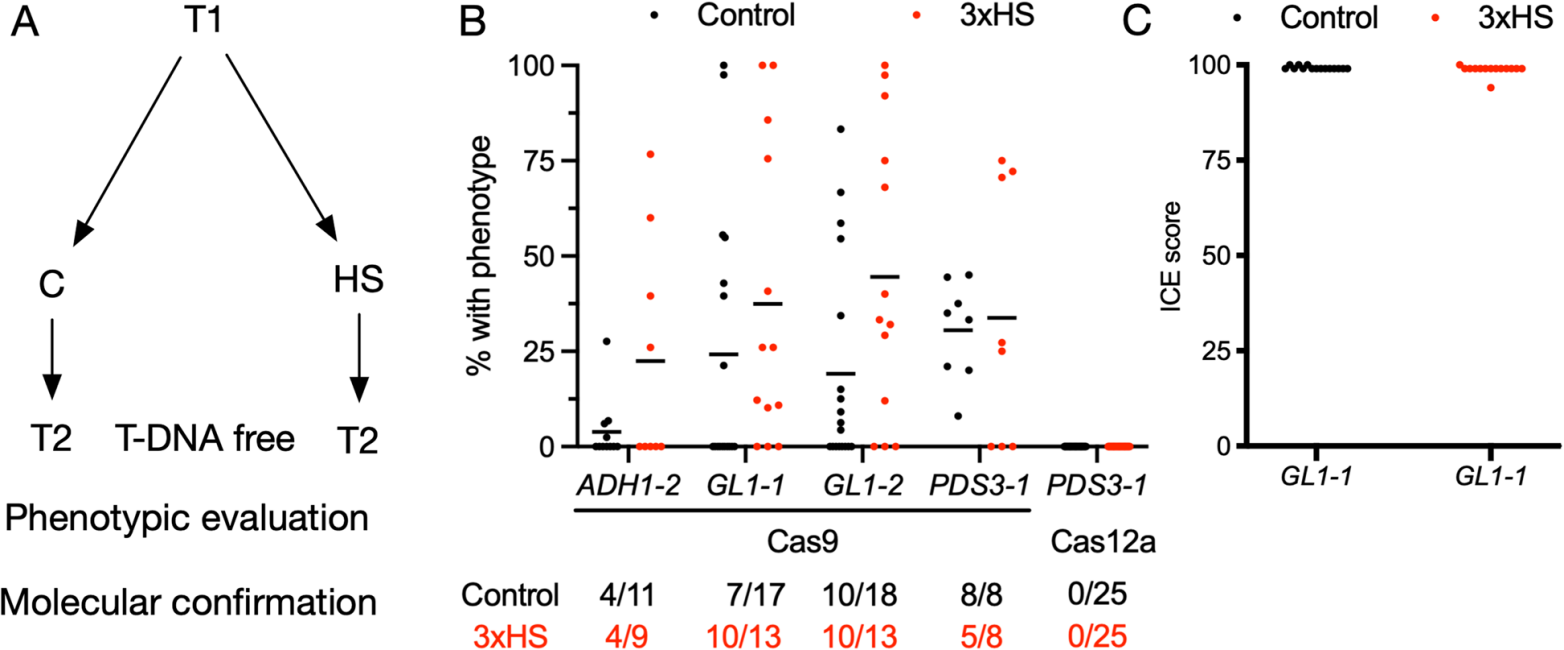
Inheritability of 3xHS induced mutations. **A** Experimental set-up to investigate inheritability of 3xHS induced mutations. **B** For each T-DNA-free T2 progeny analysed, the growth condition of T1 plants is indicated (Control or 3xHS). For each T2 progeny analysed, the percentage of plants with a *glabrous *or *pds3 *phenotype (*GL1-1*, *GL1-2* and *PDS3-1*) or displaying resistance to allyl alcohol treatment (*ADH1-2*) is indicated. *n*=25-50 per T2 plants per T1 plant. The number of T2 lines exhibiting mutant phenotypes is given for each target and condition below the graph. For Cas12a lines targeting *PDS3*, T3 progeny was scored in the same way as the T2 Cas9 lines. **C** Indel frequency in T2 lines targeting *GL1-1 *and exhibiting a *glabrous* phenotype. T1 growth conditions is indicated *n*=15 per T1 treatment

To determine if the mutations induced by heat stress can be transferred to the following generation, T1 seedlings subjected to a 3xHS or grown under control conditions were propagated to produce T2 seeds. We targeted three genes (*PDS3*, *GL1*, or *ALCOHOL DEHYDROGENASE 1* (*AT1G77120*, *ADH1*)) using Cas9. FAST-negative (T-DNA free) T2 seeds targeting *PDS3* and *GL1* were selected and grown under control conditions. Seeds from lines targeting *ADH1* were treated with allyl alcohol as plants containing wild-type alleles are killed by this treatment whereas biallelic mutants are resistant [38]. We observed that a 3xHS treatment in T1 increased the number of T2 lines with biallelic knockout phenotypes for both *GL1* targets and *ADH1-2*, but not *PDS3-1* (Fig. 4 A-B). Interestingly, the mean percentage of individuals exhibiting mutant phenotypes per line increased in the progeny of heat-treated individuals for *ADH1-2* (+19%), *GL1-1* (+13%) and *GL1-2* (+25%) compared to control (Fig. 4B). It is important to note that *PDS3* is essential for growth in the greenhouse and biallelic *pds3* mutants are sterile [39]. This negative selection may explain why the phenotypic distribution is not influenced by the heat shock in the previous generation (+3%; Fig. 4B). We confirmed biallelic mutations in individuals exhibiting a *glabrous* phenotype by Sanger sequencing (Fig. 4 C). In addition to the Cas9 targets, 50 T2 seedlings containing Cas12a and targeting *PDS3* were subjected to a 3xHS or grown under control conditions and propagated to produce T3 seeds. We did not observe *pds3* phenotypes in any FAST-negative T3 seeds grown under control conditions, indicating that the 3xHS only led to somatic mutations in these lines (Fig. 4B). In conclusion, a 3xHS treatment in the T1 generation resulted in a modest increase in the number and frequency of lines with biallelic mutations in T2, for three out of four of the Cas9 targets tested.

### Heat stress increases base editing efficiency

**Fig. 5:**
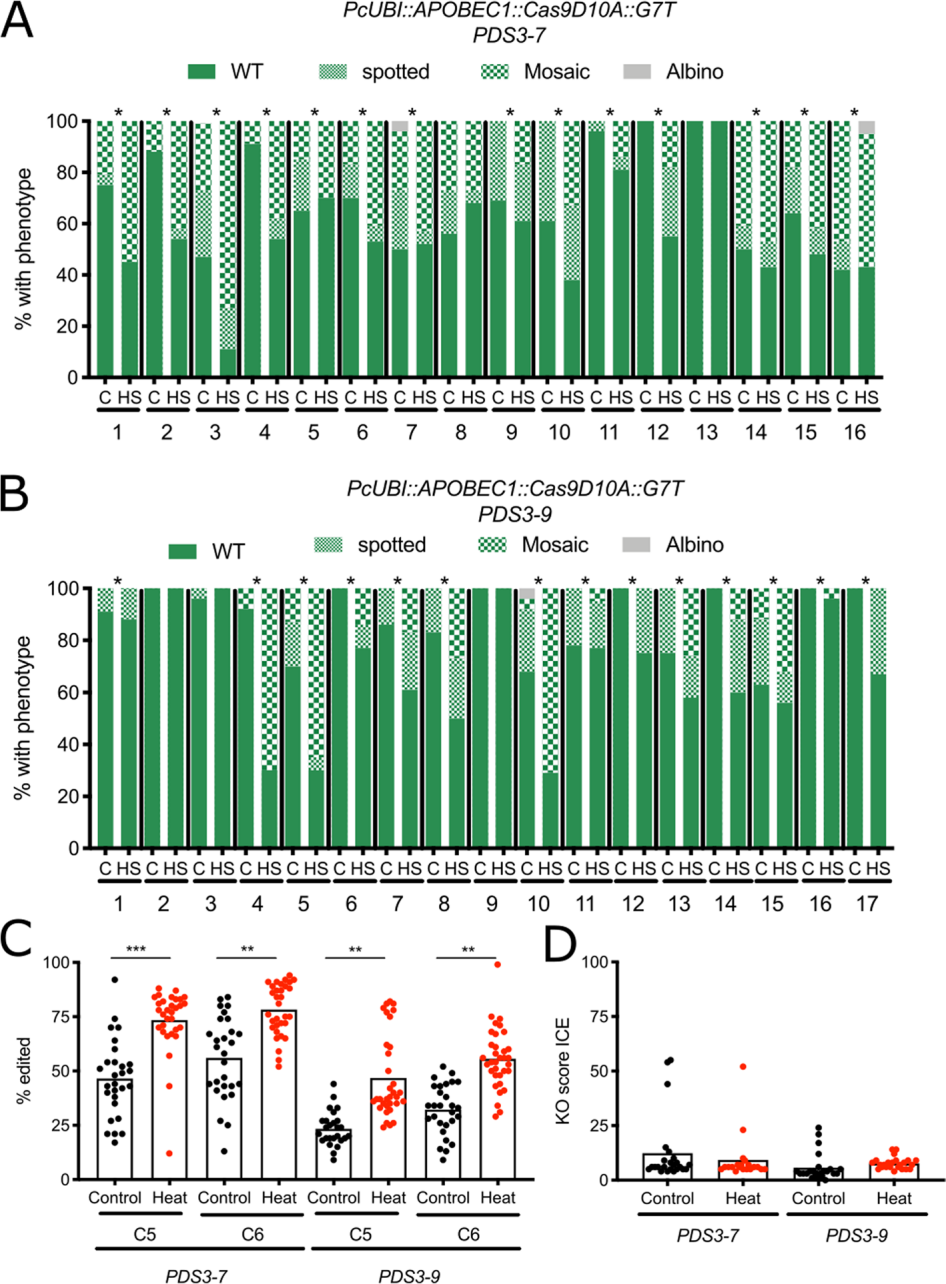
Base editing efficiency increases after heat shock. **A** Occurrence of *pds3* phenotype in 16 independent lines expressing *PcUBI::APOBEC1::Cas9D10A:G7T *and targeting *PDS3-7* grown under control (C) or heat stress (3xHS) conditions. Lines that show an increase in number and/or severity of *pds3 *phenotypes after 3xHS are indicated with *. *n*=50 per line per treatment. **B** Occurrence of *pds3* phenotype in 17 independent lines expressing *PcUBI::APOBEC::Cas9:D10A:G7T* and targeting *PDS3-9 *grown under control (C) or heat stress (3xHS) conditions. Lines that show an increase in number and/or severity of *pds3* phenotypes after 3xHS are indicated with *. *n*=50 per line per treatment. **C** Quantification of base editing. Percentage of C-to-T base editing was calculated using EditR (Kluesner et al., 2018). *n*=26-33 per target per treatment. ***: *P*<0,001; **: *P*<0,01 (One-Way ANOVA; Kruskal-Wallis test). **D** Indel frequency in base editing lines targeting *PDS3-7* or *PDS3-9*. *n*=20-24 per target per treatment

We then tested the effect of heat stress on the efficiency of base editors (Cas9 D10A nickase fused to the rat APOBEC1 cytidine deaminase (*PcUBI::APOBEC1::Cas9D10A::G7T*) [7, 40]) targeting two different locations in *PDS3* (*PDS3-7* and *PDS3-9*). This system uses APOBEC1 to deaminate cytosine (C) to uracil (U), which is predominantly repaired as a thymine (T). The *PDS3-7* and *-9* targets are designed to edit the tryptophan codon (TGG) such that base editing of any of the complementary Cs results in the generation of premature stop codon in *PDS3*. When segregating T2 lines were exposed to 3xHS, we observed an increase in the percentage and/or severity of *pds3* phenotypes in 14/16 independent lines targeting *PDS3-7* and 14/17 independent lines targeting *PDS3-*9 (Fig. 5 A-B). We confirmed that C-to-T base editing occurred at the expected position in the gRNA region (C5 and C6) using Sanger sequencing and quantifying the level of base editing using EditR [41]. For each target, we evaluated eight individuals per treatment for five independent lines. For both *PDS3* targets we observed a significant (One-way ANOVA; Kruskal-Wallis test) 22-27% increase in the percentage of C-to-T base editing, indicating that base editing is more efficient under 3xHS conditions (Fig. 5 C). In a subset of samples (27/72 for *PDS3-7* and 3/69 for *PDS3-9*) we observed C>G substitutions, but C>T was the main repair outcome and C>G editing efficiency was not affected by heat treatment. An increase in *pds3* phenotypes could also be caused by indels induced by base editors [7]. Interestingly, indel frequency was not affected by 3xHS treatment with only 6/44 heat-shocked plants containing an indel score higher than 10% (the threshold using this type of analysis) compared to 10/50 for control plants (Fig. 5D).

### Heat stress and Cas9-induced large deletions

Not all indels completely disrupt gene function. For example, exon skipping can lead to the production of aberrant, but functional, proteins [42, 43]. Therefore, large deletions are desirable to delete genomic fragments corresponding to one or several target genes. The Metacaspase (MC) gene family consists of nine members in Arabidopsis, MC1-3 belong to Type-I and MC4-9 belong to Type-II [44]. Metacaspases are cysteine-dependent proteases that induce programmed cell death. Four type-II MC genes (MC4-7) are positioned in a tandem repeat on chromosome 1 of Arabidopsis (Fig. S5). Similar to the approach by Shen et al. [45], we designed five gRNAs (A3, A9, A11, B3 and B7) to delete a 10,4 kb region from the genome. Six different constructs containing different combinations of gRNAs were generated (Fig. S5), transgenic T1 seeds were selected using FAST and then subjected to a 3xHS or control treatment. Fourteen days after the last heat treatment, the first true leaves were harvested and screened via PCR for the occurrence of large deletions. We observed 16% and 37% more plants with the expected deletion after 3xHS in lines with A3-B3 and A11-B3 gRNA combination, respectively. However, there was no clear difference for the A9-B3 and A9-B7 combinations and 18% and 21% fewer plants with the expected deletion in the A3-B7 and A11-B7 combinations, respectively (Fig. S5). We confirmed the deletions via Sanger sequencing (Fig. S5), selected six T1 individuals per line and treatment and upscaled them to T2. Twenty-five FAST-negative T2 individuals per line and treatment were screened via PCR for the deletion. Unfortunately, we did not identify any individuals with the expected large deletions, indicating that the somatic mutations were not transmitted to the progeny in the tested individuals.

### Cas9-VQR mutant

The targeting range of wild-type Cas9 is limited by the NGG protospacer-adjacent motif (PAM). Cas9 mutants with different PAMs have been identified that allow for an expansion of possible target sequences. We used the Cas9-VQR variant (D1135V/R1335Q/T1337R; *PcUBI::Cas9VQR::Pea3AT* [46, 47]) to target a specific region in the genomic region of *PEAPOD2* (*PPD2*), a regulator of leaf development [48, 49]. Cas9 PAM variants are, on average, less active compared to wild-type Cas9 [46, 50]. In agreement with this, no indels were observed when Cas9VQR plants were grown under control conditions. Two independent transgenic, single-locus T2 lines were then subjected to a 3xHS treatment or control conditions. Since no obvious plant phenotype was expected upon mutation, we genotyped the two oldest leaves in the rosette of 241 Cas9VQR plants using Sanger sequencing. We found only one individual with an indel frequency >10% at the targeted position for 121 plants grown under control conditions. In contrast, 27 out of 119 individuals had more than 50% indels rates upon 3xHS (Fig. S6). The 27 plants that received a 3xHS treatment and had the highest indel rates were upscaled to the next generation. From these T3 lines, we selected four lines that were heterozygous for the vector and genotyped 30 Cas9VQR-free plants. Unfortunately, we could not identify indels at the target site in any of the genotyped samples, indicating that the somatic mutations were not transmitted to the progeny in the tested individuals (Fig. S6).

### Heat stress and HDR efficiency

CRISPR/Cas9 efficiently induces indels via NHEJ-mediated repair in plants. In contrast, gene targeting using HDR is inefficient (<1%; [5, 51]). Since NHEJ-mediated repair and cytidine deaminases are more efficient upon 3xHS treatment, we tested if this applies to HDR as well. We used four independent segregating T2 lines expressing Cas9 fused to *LacI* (*pK LacI-Cas9*), a gRNA targeting *OLEOSIN1* (*OLE1*; *AT4G25140*) and a template containing mRuby3 and homology arms for both sides of the *OLE1* target site. Our rationale was to recreate a FAST-marker [37, 52] containing *OLE1-mRuby3* using HDR. The LacI-LacO system was utilised to interrogate whether bringing the nuclease and template in closer proximity *in vivo* could increase HDR outcomes. The four independent lines selected do not contain LacO elements, but had generated fluorescent seeds via HDR in earlier experiments [53]. Two weeks after treatment (3xHS or control), the two oldest leaves from the rosette for 19-46 individuals per line were harvested and used for PCR to detect the presence of the right border of the HDR product. Interestingly, we detected the right border in more individuals grown under control conditions compared to individuals with 3xHS treatment (Fig S7). We propagated all individuals from this experiment and determined the HDR efficiency in the next generation (T3) by counting the number of red fluorescent seeds relative to the total seed number. In line with the data obtained in the previous generation, 13 out of 122 plants from individuals grown under control conditions produced progeny with fluorescent seeds, in contrast to two out of 93 treated with 3xHS (Fig. S7). We confirmed HDR in 12 out of 13 individuals using Sanger sequencing of the right border (Fig. S7). These data indicate that HDR with our FAST reporter is less efficient after 3xHS treatment.

In conclusion, the data presented here show that 3xHS works to increase indels in Arabidopsis and Tobacco and precision modifications using base editing. The assay works on a variety of targets, appears to be independent of the regulatory sequences used to express the nuclease and is functional for Cas9 and LbCas12a. We consistently demonstrate an increase in somatic mutations but only observe an increase in inheritable mutations in some wild-type Cas9 experiments. Interestingly, our HDR reporter is negatively affected by a 3xHS.

## Discussion

CRISPR-based genome editing applications are increasingly used in plant biology to study gene function and improve germplasm (reviewed in [4]) and there is still room for optimization. CRISPR components are added or redesigned, vector assemblies are altered or growth conditions are changed to ensure high on-target genome editing efficiency (reviewed in [54]). In plants, green algae, human cell cultures and zebrafish the action of CRISPR nucleases is positively affected by heat treatment [17–19, 21, 22]. Here, we further support that heat treatment increases Cas9 and Cas12a genome editing efficiency in plants using a simple experimental set-up.

The 3xHS protocol described in this report allows one to induce a heat treatment without the need for plant growth chambers. In our hands, applying three 24 h heat treatment in a common 37 °C bacteriological incubator immediately after stratification allowed us to increase indel frequencies without losing a considerable number of plants due to stress. Furthermore, the stressed plants completed their life cycle normally when transferred to soil two weeks after the last heat cycle. Previous reports subjected Arabidopsis plants to an elevated temperature for a longer time, using four 30 h 37 °C heat cycles [17] or prolonged cultivation up to four weeks at 29 °C [18]. Our set-up takes less time, six days to complete, and results in a reliable increase in genome editing efficiency for a broad range of CRISPR targets. This works for Cas9 and LbCas12a, is independent of the promoters used to drive Cas9 expression and can result in an increased frequency of mutated alleles transmitted to the progeny. Additionally, we report that CRISPR base editing efficiency increases after a 3xHS without increasing indel frequencies. We also show that indel efficiency of Cas9 is enhanced in tobacco upon heat treatment, similar to other dicots and monocots [17, 18, 23, 25]. With this setup, 3xHS (37 °C) is the critical temperature to induce LbCas12a-mediated mutations in segregating T2 lines whereas 30 °C resulted in no obvious knockout phenotypes for the LbCas12a *PDS3* target. In contrast, cultivation at 29 °C for 14 days led to an increase in mutagenesis from background levels at two Arabidopsis targets using LbCas12a [18]. Given the low numbers of targets, different vector designs and heat treatment conditions reported to date, it is difficult to draw a strong conclusion on the exact temperature threshold. Nevertheless, heat treatment is clearly an effective way to increase the efficiency of CRISPR-based targeted genome editing applications in plants.

Applying heat treatment clearly increases somatic mutations, but researchers are often most interested in generating mutant alleles that can be transmitted to progeny for the establishment of homozygous lines. While we did observe an increase in inheritable alleles for three out of four targets tested with Cas9, we were unable to demonstrate inheritance for LbCas12a, the Cas9-VQR variant and lines generating large genomic deletions. Overall, our Cas9 results are largely consistent with those of LeBlanc et al.  [17], who demonstrated that a 37 °C heat treatment led to eight heat-treated Cas9 T1 lines giving rise to 30-100% T2s carrying mutations as compared to four controls with 0-30% mutant T2s. Our results show a greater degree of variation, with some of the control lines exhibiting 100% mutated T2s and some heat-treated lines with 0 mutated T2s. This discrepancy may in part be due to our greater sample size, or our reliance on the production of biallelic knockout mutations. Still, we do observe an increase in the average number of mutated T2 lines for three of our Cas9 targets.

The mechanism of increased CRISPR activity with a heat treatment has often been attributed to a higher nuclease activity because ~37 °C is the optimal growth temperature of the bacteria from which different Cas9 and Cas12a genes were isolated and *in vitro* experiments have shown optimal nuclease activities at this temperature [17, 21, 26, 30]. Single amino acid changes increase AsCas12a (E174R) and LbCas12a (D156R) mutagenesis efficiency twofold to sevenfold at lower temperatures in humans (25 °C versus 37 °C) and Arabidopsis (22 °C versus 28 °C), respectively [55, 56]. Since these variants were generated to alter or form new PAM proximal DNA contacts [55], temperature may influence the ability of Cas12a to access or unwind genomic DNA [19]. Nevertheless, heat stress induces a complex cellular response, integrating many signals and molecular players (reviewed in [57]) and might cause molecular changes that influence CRISPR efficiencies. For example, heat stress influences DNA repair pathways [58, 59] and cell cycle progression [60]. Importantly, heat shocking *C. reinhardtii* before Cas9-RNP delivery has been shown to increase indels rates [22] and supports a cellular state hypothesis. Furthermore, heat-treatment caused an increase in Cas9 mRNA in some wheat transformants [25], while this was not observed in Arabidopsis [17, 26]. Thus, while CRISPR enzyme dynamics may play a role in the observed heat-treatment effects, the cellular state of the target organism should be considered as well.

To further explore the potential of manipulating the cellular state, we applied different abiotic stresses known to affect DNA repair (osmotic stress, salt stress, UV-stress and DNA damage). The LbCas12a lines are well suited as marker lines for indel induction as they only display a *pds3* phenotype after 3xHS. In our hands, none of the different abiotic stress regimes were effective at inducing indels as compared to the heat treatment. Thus, heat treatment may be unique in its ability to affect CRISPR-based experiments. We think that the role of the heat-stress response on DNA repair pathways in CRISPR experiments should be more thoroughly investigated as this would help resolve the mechanism and potentially identify novel pathways or approaches to control the outcomes of genome-editing experiments.

Our results and others clearly demonstrate that a variety of CRISPR-based systems are positively affected by heat treatment: Cas9 or Cas12a indel formation using vectors or RNPs [17–19, 21, 22], prime editors [61], base editors and the Cas9-VQR variant (this report). Consequently, it is tempting to speculate that all CRISPR applications may be more efficient upon heat treatment. Our data however, demonstrate that our Cas9 HDR reporter is less efficient after heat treatment. This observation stands in contrast to findings that heat treatment positively affects HDR efficiency in tomato and can increase intrachromosomal recombination in Arabidopsis and tobacco [62–64]. Particularly interesting is the recent observation that HDR efficiency of LbCas12a, but not Cas9, increases with higher temperature in tomato [20]. We therefore suggest that more elaborate studies using more targets should assess the interplay between heat treatment and HDR-efficiency. Furthermore, the effect of heat on several CRISPR-related applications, e.g. transcriptional activation/repression, epigenetic modulation, RNA targeting and other Cas endonucleases remains to be tested.

## Conclusions

In conclusion, we present a straightforward method to increase genome editing efficiency using three heat shock cycles of 24 h separated by 24 h of recovery. Our set-up allows one to increase indel efficiency independent of target and vector system. We also observe an increase in the frequency of mutant indels transmitted to the germline. In this way, we demonstrate that heat treatment can be an easy-to-apply method to increase genome editing efficiency in a wide range of CRISPR-based applications.

## Methods

### Plant materials and growth conditions

Environmental conditions during seed production, as well as during seed storage, can affect seed vigour. Therefore, all experiments were conducted with wild-type and transgenic *Arabidopsis thaliana* (Columbia ecotype, Col-0) seeds and, within the same experiment, harvested from plants grown side by side. All transgenic lines were generated by the authors and not obtained commercially. For growth experiments, plants were grown *in vitro* on ½ Murashige and Skoog (MS) medium [65] supplemented with 1% sucrose at 21 °C under a 16-h day/8-h night regime (75µM; Spectralux Plus NL-T8 36 W/840/G13 fluorescent lamp). For stress-inducing conditions, the medium was supplemented with Mannitol (Sigma, 25mM or 50mM), NaCl (ChemLab, 50mM or 100mM), Bleomycin Sulfate (Sigma, 0,3 µg/mL) or Hydroxyurea (Sigma, 0,75mM). For the application of heat stress, plates were transferred to a common 37 °C incubator for 24 h. For UV-stress, we treated the transgenic lines with a continuous low-dose stress (40 W/m^2^ light supplemented with 0,42 W/m^2^ UV) for one, two or three days. For lines targeting *ADH1*, seeds were pre-treated with 30mM Allyl alcohol (Sigma) for two hours before sowing. All stress conditions were imposed immediately after stratification unless noted otherwise. The HDR reporter vector line pK LacI-AtCas9 was previously described [53].

### Plasmid constructs and plant transformation

All cloning reactions were transformed via heat-shock transformation into *ccd*B-sensitive DH5α *Escherichia coli* or One Shot *ccd*B Survival 2 T1R Competent Cells (Thermo Fisher Scientific). Colonies were verified via colony-touch PCR, restriction digestion, and/or Sanger sequencing by Eurofins Scientific using the Mix2Seq service. All PCR reactions for cloning were performed with Q5 High-Fidelity DNA Polymerase (New England Biolabs). Gibson assembly reactions were performed using 2× NEBuilder Hifi DNA Assembly Mix (New England Biolabs). Column and gel purifications were performed with Zymo-Spin II columns (Zymo Research). Golden Gate entry modules were constructed by PCR amplification of gene fragments and inserting the purified PCR product into a BsaI-digested GreenGate entry vector [32, 37] via restriction-ligation using BsaI (New England Biolabs) or Gibson assembly. All generated clones were verified via Sanger sequencing. See Tables S1-3 for the list of primer and target sequences, a complete list of plasmids and cloning primers.

AsCas12a, FnCas12a, and LbCas12a were amplified from pY010, pY004, and pY016, respectively and cloned into pGGC000 using restriction ligation. pY010, pY004, and pY016 were gifts from Feng Zhang (Addgene plasmids # 69,982, # 69,976, and # 69,988; [66]).

Cas12a expression vectors were assembled with Golden Gate cloning by combining pGG-A-PcUBI-B, pGG-B-Linker-C, pGG-C-Cas12a-D, pGG-D-linker-E, pGG-E-G7T-F, pGG-F-LinkerII-G into pFASTRK-AG and verified with restriction digest with PvuI and NotI. The Golden Gate destination module (A-ccdB/CmR-G) was inserted into correct plasmids via HindIII digestion and Gibson assembly as previously described [37] and confirmed with restriction digest with PvuI and NotI.

crRNA entry vectors were created using a gBlock (IDT) template containing HH and HDV ribozyme sequences with a constant Cas12a scaffold sequence and a pair of BbsI restriction sites to add novel crRNA sequences. The gBlock fragment was PCR amplified with primers flanked by BsaI restriction sites and cloned into pGGB000 via restriction ligation to create pGG-B-HH-Cas12ascaffold-HDV-C. crRNAs were designed with Geneious R11 [67] with a length of 24nt and cloned into pGG-B-HH-Cas12a-HDV-C with homolog-specific scaffolds (AsCas12a: CTTGTAGAT; FnCas12a: GTTGTAGAT; LbCas12a: AAGTGTAGAT; [66]) using annealed oligo cloning with BbsI [37]. Entry vectors were cloned into Cas12a destination vectors with pGG-A-pRPS5A-B [32], pGG-B-crRNA-C, pGG-C-linker-D, pGG-D-pea3AT-G and validated with restriction digest with NdeI.

*PDS3* Cas9 gRNAs for the promoter and tobacco tests were cloned into pEn_Chimera [68] via annealed oligo cloning. The ZmUbi, RPS5a, and 35 S Cas9 entry vectors were assembled with GoldenGate cloning as previously described for pEN-L4-PcUBI-Cas9-G7T-R1 [69]. The Cas9 entry vectors, pEn_Chimera gRNAs and pBm42GW,3 [70] were recombined with Multisite Gateway as previously described [69] and the resulting plasmids were confirmed via restriction digestion with NdeI.

pFASTRK-AtCas9-AtU6-Scaffold and pFASTGK-AtCas9-AtU6-Scaffold were created by Gateway Multisite assembly with pFASTRK24GW or pFASTGK24GW [37], pEN-L4-PcUBI-Cas9-G7T-R1, and pEN-L1-AtU6-26-BsaI-L2 [37] and confirmed with restriction digest with NdeI and HpaI. The *PDS3*, *ADH1*, *GL1-1*, *GL1-2*, *AT2G22460*, *IM1*, *VAR1-1*, *VAR1-2*, *TIR1-1* and *AT4G12990* gRNAs were added to pFASTRK-AtCas9-AtU6-Scaffold via annealed oligo cloning with BsaI and confirmed by restriction digest with NheI and Sanger sequencing of the gRNA. The large deletion vectors were made by adding paired gRNAs via a PCR approach [37] to pFASTGK-AtCas9-AtU6-Scaffold.

pFASTRK-CBE was created by performing a GoldenGate reaction with pGG-A-PcUBI-B, pGG-B-APOBEC-(GGS)5-C [40], pGG-C-Cas9-D10A-D, pGG-D-UGI-NLS-E, pGG-E-G7T-F, pGG-F-AtU6-26-AarI-AarI-G into pFASTRK-AG and the AarI site replaced with BsaI-ccdB/CmR-BsaI as previously described [37]. The *PDS3-7* and *PDS3-9* gRNAs were added to pFASTRK-CBE via annealed oligo cloning with BsaI and confirmed by restriction digest with NheI and BamHI and Sanger sequencing of the gRNA.

The *PPD2* gRNAs were added to a Cas9-VQR variant as previously described [47].

Plant vectors were transformed in *Agrobacterium tumefaciens* C58C1 by electroporation and transformation of *Arabidopsis thaliana* was performed via floral-dip [71]. For the construct containing the FASTR or FASTG screenable marker [37], T1 transgenic seeds were selected under a fluorescence stereomicroscope (Leica M165FC). Non-fluorescent T2 lines were assumed to be T-DNA free. Unless specified otherwise, segregating T2 lines used in growth experiments were not checked for homozygosity or single-locus insertion of the transgene. Transformation of *Nicotiana tabacum SR-1* was done by cocultivation of leaf explants with *Agrobacterium* [72].

### DNA extraction, PCR and sequencing analyses

Plant material was harvested for DNA extraction with the CTAB method [73]. Either the first true leaf pairs or entire seedlings were harvested, depending if the material was upscaled or not. A region around the CRISPR/Cas target site was PCR amplified using ALLin Red Taq Master Mix, 2X (highQu). The PCR products were analysed via agarose gel electrophoresis and purified by bead purification with HighPrep PCR (MAGBIO). The purified samples were sent for Sanger sequencing (Mix2seq; Eurofins Scientific) and analysed using ICE (https://ice.synthego.com/#/) and/or EDITR [41]. The ICE KO-score represents the proportion of cells that have either a frameshift or 21+ bp indel. See Supporting Tables for the list of primer and target sequences. The number of individuals analysed is specified for each experiment.

## Supplementary Information

**Additional file 1: Figure S1.** Phenotypic effect of 3xHS treatment. (A) Phenotype of soil-grown segregating *PcUBI::LbCas12a::G7T* and *PcUBI::Cas9::G7T* lines under control conditions or 3xHS regime. Plants displaying a *pds3 *phenotype are indicated with a white circle. (B) T2 plants containing AsCas12a, FnCas12a, LbCas12a or Cas9 and targeting *PDS3*. DNA was extracted for three to twelve individuals for each line and treatment, PCR products amplified from targeted loci were sequenced and analysed using ICE (https://ice.synthego.com). The KO-score is given for each sample, indicating indels that result in a frameshift or are 21+bp in length. Lines indicate mean KO-score per sample per treatment. n=3-12 per sample per treatment. (C) Phenotype of *in vitro *grown segregating *PcUBI::Cas9::G7T* and *PcUBI::LbCas12a::G7T* lines under control conditions or 3xHS regime. Picture taken 14 days after the last heat shock. White arrowheads indicate *PcUBI::LbCas12a::G7T *plants displaying *pds3 *phenotype after 3xHS. **Figure S2.** Critical temperature to induce genome editing. (A) Phenotypic effect of number of consecutive heat shocks on segregating Arabidopsis T2 lines expressing *PcUBI::LbCas12a::G7T* (two independent lines, Cas12a #3-#4) and a gRNA targeting *PDS3*. Transgenic lines and wild-type control (WT) were grown under control conditions (21 Phenotypic effect of number of consecutive heat shocks on segregating Arabidopsis T2 lines expressing *PcUBI::LbCas12a::G7T* (two independent lines, Cas12a #3-#4) and a gRNA targeting *PDS3*. Transgenic lines and wild-type control (WT) were grown under control conditions (21°C) or subjected to one, two, three or four heat shocks (1-4xHS; 37°C). Pictures taken 14 days after recovery. (C) Phenotypic distribution of wild-type (WT) and two independent transgenic lines containing *PcUBI::LbCas12a::G7T* subjected to control (C) conditions or 3xHS treatments at 30°C, 37°C or 42°C. N.G.: Not Germinated. n=100 per line per treatment. **Figure S3.** Effect of other stress conditions on genome editing efficiency. Segregating Arabidopsis T2 lines expressing *PcUBI::LbCas12a::G7T* (two independent lines) and a gRNA targeting *PDS3* were grown under control conditions (21°C) on 1/2MS medium supplemented with Bleomycin (0,3mg/ml), Hydroxyurea (HU; 0,75mM), Mannitol (25mM or 50mM) or NaCl (50mM or 100mM). Additionally, transgenic lines were exposed to one, two or three 24h cycles of 40 W/m^-2^ light supplemented with 0,42 W/m^-2^ UV alternated with 24h of control light conditions. n=50 for each line and treatment. **Figure S4.** Heat-induced genome editing is independent of the transcriptional regulator. T2 segregating lines of four constructs (*pB-ZmUBI-Cas9PTA-G7T, pB-RPS5PA-Cas9PTA-G7T, pB-35SP-Cas9PTA-G7T *and* pB-PcUBI-Cas9PTA-G7T*) targeting *PDS3* with one of four possible gRNAs) were grown under control conditions (C) or subjected to 3xHS (HS). *pds3* phenotypes were scored according to the severity (albino, mosaic or spots) 14 days after final HS for each line and condition. Lines that show an increase in number and/or severity of *pds3* phenotypes after 3xHS are indicated with *. n=25 for each line and condition. **Figure S5.** Effect of 3xHS on inducing large deletions. (A) Experimental set-up. Four type-II MC genes (MC4-7) are positioned in a tandem repeat on chromosome 1. We designed five gRNAs (A3, A9, A11, B3 and B7) to delete the 10,4kB region from the genome. (B) Six independent lines with different combinations of gRNAs were submitted to 3xHS (HS) or control treatment (C). 14 days after the last HS, plants were genotyped. The number of plants where the intended deletion was observed is indicated as a ratio (relative to the number of plants genotyped). (C) Molecular confirmation of deletion. For each independent line, the PCR product corresponding to a big deletion was sequenced for 4-6 individuals. Sequence reads were mapped to the expected deletions using the Geneious software. Alignments are shown for those lines where a single repair product was observed in the PCR product. (D) Example of the genotyping strategy. A11-B7 T2 lines grown under control conditions (in this example), an untransformed control (WT) and a no-template control (-) were genotyped with two pairs of primers: one control reaction amplifying a part of *AtMC7* (ATMC7-FW + ATMC7-RV) and one reaction amplifying the intended deletion (ATMC7-FW + ATMC4-RV; see also panel A). **Figure S6.** Effect of 3xHS on Cas9-VQR activity. Two independent transgenic single-locus T2 lines containing *PcUBI::Cas9VQR::Pea3AT* were imposed to a 3xHS treatment or control conditions. Two weeks after the final heat shock, the two oldest leaves were genotyped by Sanger sequencing. n=121 (T2 Control), 119 (T2 Heat) or 30 (T3). **Figure S7.** Effect of 3xHS on HDR efficiency. (A) Experimental set-up to insert mRuby3 at the C-terminus of *OLE1*(*AT4G25140*). Schematic representation of the T-DNA constructs (pK LacI-AtCas9; top), the endogenous *OLE1* locus (middle) and *OLE1-mRuby3* HR product (bottom). Abbreviations: right border (RB), left border (LB), G7 terminator (G7T), kanamycin resistance cassette (nptII), nopaline synthase terminator (NosT). (B) Results of genotyping PCR two weeks after the last heat shock of T2 lines expressing *Cas9* fused to *LacI*, a gRNA targeting *OLE1* and a template containing mRuby and overhang sequences at both sides of the target region. For each line and condition (control or 3xHS), the number of plants where the right border of the insertion was amplified relative to the total number of plants genotyped is shown. A representative example is shown above the table; lines where the right border was amplified are indicated with a black arrow. (C) Left: Visual confirmation of putative HDR events in T3 lines. A functional OLE1-mRuby construct results in red fluorescent seeds, indicated with white arrows. Right: Quantification of HR efficiency in three independent T3 lines that were subjected to control conditions or 3xHS in the previous generation. Each dot indicated the HR efficiency per line per treatment, the number of lines with visual observation of HR relative to total number of screened lines is indicated below the graphs. (D) Molecular confirmation of HR events in 13 independent T3 individuals. The right border sequence was amplified, sequenced and mapped to the expected HR outcome using the Geneious software. Black bars indicate disagreements to the reference sequence.

**Additional file 2: Table S1.** List of targets per vector construct. **Table S2.** Complete list of generated vectors (entry, destination and expression). **Table S3.** List of cloning primers and gBlock sequence.

## Data Availability

All data generated or analysed during this study are included in this published article [and its supplementary information files]. All plasmid DNA sequences generated for this study (Table S2) are available on the VIB-UGent plasmid repository https://gatewayvectors.vib.be.
