# Supplementary material for: The heat is on: a simple method to increase genome editing efficiency in plants
Source: BMC Plant Biol. 2022 Mar 24;22:142. doi: 10.1186/s12870-022-03519-7 (PMC8951696; doi:10.1186/s12870-022-03519-7)
Supplement: Supplementary file 1 — Additional file 1: Figure S1. Phenotypic effect of 3xHS treatment. (A) Phenotype of soil-grown segregating PcUBI::LbCas12a::G7T and PcUBI::Cas9::G7T lines under control conditions or 3xHS regime. Plants displaying a pds3 phenotype are indicated with a white circle. (B) T2 plants containing AsCas12a, FnCas12a, LbCas12a or Cas9 and targeting PDS3. DNA was extracted for three to twelve individuals for each line and treatment, PCR products amplified from targeted loci were sequenced and analysed using ICE (https://ice.synthego.com). The KO-score is given for each sample, indicating indels that result in a frameshift or are 21+bp in length. Lines indicate mean KO-score per sample per treatment. n=3-12 per sample per treatment. (C) Phenotype of in vitro grown segregating PcUBI::Cas9::G7T and PcUBI::LbCas12a::G7T lines under control conditions or 3xHS regime. Picture taken 14 days after the last heat shock. White arrowheads indicate PcUBI::LbCas12a::G7T plants displaying pds3 phenotype after 3xHS. Figure S2. Critical temperature to induce genome editing. (A) Phenotypic effect of number of consecutive heat shocks on segregating Arabidopsis T2 lines expressing PcUBI::LbCas12a::G7T (two independent lines, Cas12a #3-#4) and a gRNA targeting PDS3. Transgenic lines and wild-type control (WT) were grown under control conditions (21 Phenotypic effect of number of consecutive heat shocks on segregating Arabidopsis T2 lines expressing PcUBI::LbCas12a::G7T (two independent lines, Cas12a #3-#4) and a gRNA targeting PDS3. Transgenic lines and wild-type control (WT) were grown under control conditions (21°C) or subjected to one, two, three or four heat shocks (1-4xHS; 37°C). Pictures taken 14 days after recovery. (C) Phenotypic distribution of wild-type (WT) and two independent transgenic lines containing PcUBI::LbCas12a::G7T subjected to control (C) conditions or 3xHS treatments at 30°C, 37°C or 42°C. N.G.: Not Germinated. n=100 per line per treatment. Figure S3. Effect of other stress [file 12870_2022_3519_MOESM1_ESM.docx]

The following Supporting Information is available for this article:

**Figure S1.** Phenotypic effect of 3xHS treatment.

**Figure S2.** Critical temperature to induce genome editing.

**Figure S3.** Effect of other stress conditions on genome editing efficiency.

**Figure S4.** Heat-induced genome editing is independent of the transcriptional regulator.

**Figure S5.** Effect of 3xHS on inducing large deletions.

**Figure S6.** Effect of 3xHS on Cas9-VQR activity.

**Figure S7.** Effect of 3xHS on HDR efficiency.

**Table S1.** List of targets per vector construct.

**Table S2.** Complete list of generated vectors (entry, destination and expression).

**Table S3.** List of cloning primers and gBlock sequence.

**Figure S1.** **Phenotypic effect of 3xHS treatment.**

(A) Phenotype of soil-grown segregating *PcUBI::LbCas12a::G7T* and *PcUBI::Cas9::G7T* lines under control conditions or 3xHS regime. Plants displaying a *pds3* phenotype are indicated with a white circle.
(B) T2 plants containing AsCas12a, FnCas12a, LbCas12a or Cas9 and targeting *PDS3*. DNA was extracted for three to twelve individuals for each line and treatment, PCR products amplified from targeted loci were sequenced and analysed using ICE (https://ice.synthego.com). The KO-score is given for each sample, indicating indels that result in a frameshift or are 21+bp in length. Lines indicate mean KO-score per sample per treatment. n=3-12 per sample per treatment.
(C) Phenotype of *in vitro* grown segregating *PcUBI::Cas9::G7T* and *PcUBI::LbCas12a::G7T* lines under control conditions or 3xHS regime. Picture taken 14 days after the last heat shock. White arrowheads indicate *PcUBI::LbCas12a::G7T* plants displaying *pds3* phenotype after 3xHS.

**Figure S2. Critical temperature to induce genome editing.**

(A) Phenotypic effect of number of consecutive heat shocks on segregating Arabidopsis T2 lines expressing *PcUBI::LbCas12a::G7T* (two independent lines, Cas12a #3-#4) and a gRNA targeting *PDS3*. Transgenic lines and wild-type control (WT) were grown under control conditions (21°C) or subjected to one, two, three or four heat shocks (1-4xHS; 37°C). Pictures taken 14 days after recovery.
(B) Phenotypic distribution of wild-type (WT) and two independent transgenic lines containing *PcUBI::LbCas12a::G7T* subjected to control (C) conditions or 3xHS treatments at 30°C, 37°C or 42°C. N.G.: Not Germinated. n=100 per line per treatment.

**Figure S3. Effect of other stress conditions on genome editing efficiency.**

Segregating Arabidopsis T2 lines expressing *PcUBI::LbCas12a::G7T* (two independent lines) and a gRNA targeting *PDS3* were grown under control conditions (21°C) on 1/2MS medium supplemented with Bleomycin (0,3μg/ml), Hydroxyurea (HU; 0,75mM), Mannitol (25mM or 50mM) or NaCl (50mM or 100mM). Additionally, transgenic lines were exposed to one, two or three 24h cycles of 40 W/m^-2^ light supplemented with 0,42 W/m^-2^ UV alternated with 24h of control light conditions. n=50 for each line and treatment.

**Figure S4. Heat-induced genome editing is independent of the transcriptional regulator.**

T2 segregating lines of four constructs (*pB-ZmUBI-Cas9PTA-G7T, pB-RPS5PA-Cas9PTA-G7T, pB-35SP-Cas9PTA-G7T* and *pB-PcUBI-Cas9PTA-G7T*) targeting *PDS3* with one of four possible gRNAs) were grown under control conditions (C) or subjected to 3xHS (HS). *pds3* phenotypes were scored according to the severity (albino, mosaic or spots) 14 days after final HS for each line and condition. Lines that show an increase in number and/or severity of *pds3* phenotypes after 3xHS are indicated with *. n=25 for each line and condition.

**Figure S5. Effect of 3xHS on inducing large deletions.**

(A) Experimental set-up. Four type-II MC genes (MC4-7) are positioned in a tandem repeat on chromosome 1. We designed five gRNAs (A3, A9, A11, B3 and B7) to delete the 10,4kB region from the genome.
(B) Six independent lines with different combinations of gRNAs were submitted to 3xHS (HS) or control treatment (C). 14 days after the last HS, plants were genotyped. The number of plants where the intended deletion was observed is indicated as a ratio (relative to the number of plants genotyped).
(C) Molecular confirmation of deletion. For each independent line, the PCR product corresponding to a big deletion was sequenced for 4-6 individuals. Sequence reads were mapped to the expected deletions using the Geneious software. Alignments are shown for those lines where a single repair product was observed in the PCR product.
(D) Example of the genotyping strategy. A11-B7 T2 lines grown under control conditions (in this example), an untransformed control (WT) and a no-template control (-) were genotyped with two pairs of primers: one control reaction amplifying a part of *AtMC7* (ATMC7-FW + ATMC7-RV) and one reaction amplifying the intended deletion (ATMC7-FW + ATMC4-RV; see also panel A).

**Figure S6. Effect of 3xHS on Cas9-VQR activity.**

Two independent transgenic single-locus T2 lines containing *PcUBI::Cas9VQR::Pea3AT* were imposed to a 3xHS treatment or control conditions. Two weeks after the final heat shock, the two oldest leaves were genotyped by Sanger sequencing. n=121 (T2 Control), 119 (T2 Heat) or 30 (T3).

**Figure S7. Effect of 3xHS on HDR efficiency.**

(A) Experimental set-up to insert mRuby3 at the C-terminus of *OLE1* (*AT4G25140*). Schematic representation of the T-DNA constructs (pK LacI-AtCas9; top), the endogenous *OLE1* locus (middle) and *OLE1-mRuby3* HR product (bottom). Abbreviations: right border (RB), left border (LB), G7 terminator (G7T), kanamycin resistance cassette (nptII), nopaline synthase terminator (NosT).
(B) Results of genotyping PCR two weeks after the last heat shock of T2 lines expressing *Cas9* fused to *LacI*, a gRNA targeting *OLE1* and a template containing mRuby and overhang sequences at both sides of the target region. For each line and condition (control or 3xHS), the number of plants where the right border of the insertion was amplified relative to the total number of plants genotyped is shown. A representative example is shown above the table; lines where the right border was amplified are indicated with a black arrow.
(C) Left: Visual confirmation of putative HDR events in T3 lines. A functional OLE1-mRuby construct results in red fluorescent seeds, indicated with white arrows. Right: Quantification of HR efficiency in three independent T3 lines that were subjected to control conditions or 3xHS in the previous generation. Each dot indicated the HR efficiency per line per treatment, the number of lines with visual observation of HR relative to total number of screened lines is indicated below the graphs.
(D) Molecular confirmation of HR events in 13 independent T3 individuals. The right border sequence was amplified, sequenced and mapped to the expected HR outcome using the Geneious software. Black bars indicate disagreements to the reference sequence.
